# Breaking down the laughter: an exploration into the linguistic dimensions in stand-up comedy ratings

**DOI:** 10.1186/s40359-024-02187-6

**Published:** 2024-11-20

**Authors:** Herald Cela, Sarah-Vanessa Veit, Guilherme Wood

**Affiliations:** 1 Department of Psychology, University of Graz, Graz, Austria; 2 BioTechMed-Graz, Graz, Austria

**Keywords:** Stand-up comedy, Linguistic analysis, Humor production, Audience reception, Social processes, Affect

## Abstract

Despite the pervasive influence of humor in society, comprehensive quantitative studies exploring its dimensions and societal reception are scarce. This study advances the field by analyzing stand-up comedy specials, linking linguistic elements to humor theories and audience ratings, using the Linguistic Inquiry and Word Count (LIWC-22). The analysis explored the presence and association of Affect, Social, and Cognitive dimensions with ratings of standup comedy shows. Statistical analyses entailed using linear mixed models, incorporating comedians as random effects. Female comedians consistently received lower ratings than males, reflecting historical gender biases, while recent shows received lower ratings than older ones, suggesting the challenge of surpassing pioneering works. Negative emotion positively influenced ratings, while negative mood words had a detrimental effect, highlighting the delicate balance in comedic narratives. Social markers significantly predicted ratings, with moralization negatively impacting them, while the use of social referents, particularly female pronouns, improved ratings. These findings offer valuable insights into the linguistic dynamics of stand-up comedy, informing future research and practice in the comedy industry.

**Supplementary Information:**

The online version contains supplementary material available at 10.1186/s40359-024-02187-6.

## Background

Laughter serves as a universally recognized expression of joy and amusement, often facilitated by humor. Research into humor has highlighted its psychological and social benefits, such as enhancing social bonds and providing emotional relief [1, 2]. Despite the widespread recognition of these benefits, the mechanisms underlying what makes something humorous remain a topic of ongoing debate. Understanding these mechanisms is essential for exploring how humor operates in different contexts, such as stand-up comedy, which utilizes various elements to create comedic effects. This study aims to examine these elements by analyzing the language used in stand-up comedy, using linguistic markers to better understand how humor functions in this specific form of entertainment.

Various theories have emerged in the quest to understand the nature of humor, with the most classic and referenced being the incongruity theory, the superiority theory, and the relief theory [3–5]. The incongruity theory suggests that humor arises from disparities between our expectations and outcomes [3, 4]. This framework includes cognitive components, where discrepancies at different levels of significance may generate ambiguity, thus leading to humor. Comedians often subvert anticipated outcomes with punchlines, resulting in unexpected and humorous effects. The superiority theory posits that humor stems from a sense of superiority towards others [5, 6]. Social processes may be at play following this perspective, leveraging stereotypes to construct in-group to out-group dynamics. For example, this entails using satire and parody to challenge established norms and conventions by critiquing political leaders and institutions [7, 8]. The relief theory contends that humor emerges from tension release [9, 10], often through taboo subjects. Affective processes such as stress relief may partly explain humor as the release of negative energies. Instances of this theory include jokes that confront uncomfortable subjects, such as sexual fiascos. More recently, the benign-violation theory (BVT) combines previous perspectives by suggesting that humor occurs when a situation simultaneously violates social norms or expectations while being perceived as benign or non-threatening by the observer. According to this theory, for a violation to be considered humorous, it must be interpreted as harmless, which can depend on factors like psychological distance, context, or a shared understanding between the comedian and the audience [7].

Although the BVT has gathered some evidence to support its validity, it is important to acknowledge the extensive body of research that has explored the linguistic and semantic aspects of humor. Theories such as the General Theory of Verbal Humor and studies on lexical priming and semantic prosody have provided significant insights into how humor is constructed and understood [11, 12]. Lexical priming theory, for instance, suggests that humor may arise from the way certain words activate related concepts in the mind, contributing to unexpected or incongruent meanings that lead to comedic effects. Semantic prosody, on the other hand, focuses on how the emotional or evaluative associations of words, especially in recurring contexts, contribute to the overall tone of humor, which is often manipulated in comedy to subvert audience expectations. However, despite these advances, there remains a gap in understanding the specific linguistic markers that contribute to humor appreciation in stand-up comedy, especially when analyzed through the lens of a more psychological perspective and contemporary computational methods.

Advancements in automatic text analysis have revolutionized the study of entertainment and artistic expression from a linguistic standpoint, providing researchers with objective and comprehensive tools. Both machine learning algorithms and dictionary-based approaches have enabled the prediction of the emotional impact of words as well as their structured organization in corpora [13]. These tools can be tuned to incorporate predictors to observe their different effects on linguistic expression, including gender and age [14]. It would, therefore, be particularly interesting to draw comparisons between linguistic markers used in generating humor versus those used in daily conversation. In addition, text analysis techniques can also be instrumental in examining the effect of the language used by stand-up comedians in the reception of humor. By scrutinizing audience reception to different types of humor, researchers can gain insights into the factors that influence comedic preferences and the dynamics of comedic performances [15]. Notably, some studies have used similar approaches to explore gender-related linguistic disparities, offering partial insights into humor usage and the reception of comedic acts [16–18]. However, these studies have focused on broad linguistic and emotional patterns, and little work has specifically addressed how these methods can be applied to analyze the specific linguistic features of stand-up comedy. This underscores the need for more targeted approaches that integrate both computational and psychological insights to understand humor in this art form.

Stand-up comedy serves as a contemporary and prominent outlet for humor, characterized by its unique artistic expression heavily reliant on linguistic style, timing, and delivery to evoke laughter from audiences. However, despite its widespread popularity, scholarly humor research on stand-up comedy remains in its early stages [19]. While there has been substantial research on humor in broader contexts, including theoretical and computational approaches [11, 20], studies specifically addressing stand-up comedy’s linguistic markers and their relationship with audience reception are relatively limited. The few available studies (e.g. [11, 15]) indicate that language in stand-up comedy operates differently from other comedic formats due to its live nature and direct interaction with audiences. Therefore, understanding the specific linguistic features in this setting presents a distinct challenge that merits further investigation.

Our study aims to fill these gaps by investigating these differences among comedians while considering essential variables such as gender, age, and period of time. To achieve this, we collected and analyzed transcripts of American stand-up comedy shows using automatic text processing with a dictionary-based approach.

Guided by theoretical foundations in humor, we hypothesize that stand-up comedy shows will exhibit higher frequencies of terms related to cognition, affect, and social processes compared to general speech. These categories capture key elements often manipulated in humor to produce comedic effects. For example, cognitive terms reflect the surprise or incongruity common in punchlines, aligning with incongruity theory, which posits that humor emerges from unexpected discrepancies between expectations and reality. Social terms highlight relational dynamics, supporting superiority theory, where humor arises from a sense of triumph over others. Affective terms convey emotional tension and release, consistent with relief theory, in which humor serves to alleviate psychological stress. Unlike other art forms, stand-up comedy frequently relies on these cognitive, social, and emotional manipulations to elicit laughter and engage the audience in a humorous experience. Additionally, we also aim to investigate the impact of these processes on the reception of comedic performances. We anticipate that all three categories (cognitive, affect, social processes) will significantly contribute to predicting ratings of the show.

While our study utilizes a dictionary-based approach to analyze linguistic patterns in stand-up comedy, it is important to emphasize that this method is primarily descriptive. The goal is to identify correlations between linguistic markers and humor appreciation, providing a foundation for further investigation into these patterns rather than asserting definitive causal explanations for humor. We expect that our findings will provide insights into the role of language in comedic performances and contribute to our understanding of the underlying mechanisms that influence the popularity and reception of stand-up comedy acts.

## Methods

### Sample

To compile our dataset, we systematically gathered transcripts of stand-up comedy shows from openly accessible online sources, adhering to specific inclusion criteria. These criteria stipulated that the content must be in English, the comedian must be active in the United States, each comedian must have a minimum of two shows available, and the duration of each show must fall between 30 min and 90 min. These criteria aimed to ensure a balanced and diverse dataset while controlling for variability among comedians and generating substantial transcript content. Additionally, the geographic constraint helped mitigate potential influences of varied cultural contexts on linguistic markers.

The majority of the transcripts were sourced from a comprehensive repository of publicly accessible comedy specials [21], last accessed on July 25, 2023. Given the public nature of these transcripts, the names of the comedians and the titles of the shows were disclosed, allowing for the identification of individual participants. It’s important to note that our research exclusively utilized publicly available information, adhering to ethical research standards and maintaining privacy and confidentiality. As such, no specific ethics approval was sought nor informed consent obtained, as the data were obtained from a publicly accessible source intended for educational and non-profit purposes only, and used in accordance with the terms of use specified by the website.

Due to the limited availability of shows by female comedians, we employed an extensive exploratory approach to gather all available material in this category. To ensure comparable sample sizes, we randomly selected transcripts from male comedians to match the volume collected from their female counterparts. In cases where only one transcript was accessible per comedian, additional specials were manually transcribed from video clips on platforms like YouTube, ensuring that each comedian had at least two specials for thorough analysis.

Our final dataset comprised 153 transcripts from 48 comedians, including 26 male and 22 female comedians. Additionally, we collected supplementary variables for each show, such as production year, the comedian’s age at the time of performance, and the show’s IMDb rating, sourced from publicly available web sources [22, 23]. We treated the production year as a continuous variable in our analysis, allowing us to assess whether there is a general trend of increasing or decreasing ratings over time. IMDb ratings, scored from 1 to 10 with decimal values, were standardized to account for variations in the number of voters, ensuring consistency in rating comparisons across shows. This standardization was essential for accurately weighing the ratings based on the number of users who had voted.

### Instruments and measures

To dissect the linguistic features embedded within stand-up comedy transcripts, we employed the Linguistic Inquiry and Word Count (LIWC) software, a computational tool designed to categorize words based on their psychological and emotional connotations. With an extensive dictionary spanning over 12,000 words, LIWC classifies language based on various linguistic attributes, including affective, social, cognitive, and perceptual dimensions. This instrument, validated on extensive populations, offers reliability values (*Cronbach’s Alphas*) for each linguistic category, providing researchers with confidence in its accuracy and consistency. Scholarly investigations have affirmed that linguistic style, as captured by LIWC, tends to be an enduring individual trait that maintains consistency across diverse temporal and situational contexts [24]. Furthermore, research has demonstrated correlations between linguistic style and socio-demographic variables such as age [18] and gender [25].

For our analysis, we employed the most up-to-date version of the software, LIWC-22 [26], equipped with linguistic categories tailored to detect elements of cognition, social interaction, and affect. These categories align closely with our research goals, allowing us to capture the distinct linguistic features present in stand-up comedy transcripts. To identify cognitive elements, LIWC offers a *Cognition* macro-category, along with nine subcategories that specify different cognitive processes. These categories were chosen for their ability to detect the unexpectedness and cognitive dissonance inherent in humor. For example, humor frequently involves surprising punchlines or incongruous situations that challenge the audience’s expectations, creating a moment of cognitive dissonance. The cognitive processes captured by these LIWC categories, such as insight, causation, and discrepancy, are reflective of the mental adjustments the audience makes to resolve this dissonance and find humor in the unexpected. Similarly, the *Social processes* macro-category, with its 11 subcategories, was selected to capture the social elements prevalent in humor, as comedians often reference others in their performances. Lastly, the *Affect* macro-category, divided into nine subcategories, was chosen to capture words related to moods and emotions commonly found in humor. This aligns with theories of relief, which suggest that humor can function as a way to release negative emotions like anxiety or anger and evoke positive emotions. Literature supports the idea that humor can involve a range of emotions, from the expression of joy and happiness to the relief of negative feelings like sadness or anxiety [1, 27].

To compare the linguistic markers found in stand-up comedy shows with those from more typical forms of communication, we utilized the population norms provided in the LIWC manual [28]. These norms, specifically from the ‘natural conversations’ category, are based on a broad sampling of spoken language that reflects everyday discourse. The ‘natural conversations’ dataset includes transcripts from a variety of informal spoken interactions, such as personal conversations, interviews, and spontaneous dialogues. This dataset is part of the LIWC’s broader ‘kitchen test’ approach, which aims to create a comprehensive benchmark by including a diverse range of text types and contexts.

Given the descriptive nature of the LIWC tool, this study aims to explore potential correlations between specific linguistic markers and humor appreciation in stand-up comedy shows. Our analysis should be viewed as hypothesis-generating, providing a basis for further research rather than definitive explanations.

A detailed overview of the selected LIWC categories, including examples, dictionary entries, and *Cronbach’s Alpha* values, can be found in Table 1.

**Table 1:** Selected LIWC categories

Category	Examples	Entries	Alpha
Cognition	is, was, but, are	1403	0.68
All-or-none	all, no, never, always	35	0.37
Cognitive processes	but, not, if, or, know	1365	0.67
Insight	know, how, think, feel	383	0.43
Causation	how, because, make, why	169	0.21
Discrepancy	would, can, want, could	108	0.29
Tentative	if, or, any, something	230	0.52
Certitude	really, actually, of course, real	131	0.22
Differentiation	but, not, if, or	325	0.38
Memory	remember, forget, remind, forgot	26	0.23
Affect	good, well, new, love	2999	0.64
Positive tone	good, well, new, love	1020	0.61
Negative tone	bad, wrong, too much, hate	1530	0.62
Emotion	good, love, happy, hope	1030	0.61
Positive emotion	good, love, happy, hope	337	0.52
Negative emotion	bad, hate, hurt, tired	618	0.52
Anxiety	worry, fear, afraid, nervous	120	0.37
Anger	hate, mad, angry, frustr*	181	0.30
Sadness	sad, disappoint*, cry	134	0.25
Swear words	shit, fuckin*, fuck, damn	462	0.79
Social processes	you, we, he, she	2760	0.43
Social behavior	said, love, say, care	1632	0.49
Prosocial behavior	care, help, thank, please	242	0.49
Politeness	thank, please, thanks, good morning	142	0.58
Interpersonal conflict	fight, kill, killed, attack	305	0.43
Moralization	wrong, honor*, deserv*, judge	356	0.37
Communication	said, say, tell, thank*	350	0.42
Social referents	you, we, he, she	1232	0.35
Family	parent*, mother*, father*, baby	194	0.48
Friends	friend*, boyfriend*, girlfriend*, dude	102	0.27
Female references	she, her, girl, woman	254	0.56
Male references	he, his, him, man	230	0.62

Note: The table displays all the LIWC categories selected for our analyses, relevant for both humor production and appreciation. The “Entries” column expresses the size of terms included in the dictionary, while the “Alpha” column represents the reliability analyses conducted for each category during validation studies [28]

### Pre-processing and analyses

Before subjecting the transcripts to LIWC’s analytical scrutiny, we undertook several pre-processing steps to refine the data. This involved the removal of unnecessary passages, such as text enclosed within brackets, musical notes, and song-related content. These sections, often containing audience reactions like applause and laughter or song lyrics, held no relevance to our analysis. Subsequently, we compiled the refined text into a continuous format, eliminating paragraph breaks, and stored it in .*txt* files for further processing through the LIWC software.

The output generated by LIWC is in the form of a structured table, with each row representing a specific transcript and each column delineating a distinct LIWC category. This organized format facilitated further statistical analysis and visualization using dedicated software.

### Statistical analysis

We constructed a separate linear mixed model (LMM) for each LIWC variable, treating each variable as an independent response variable. This approach allowed us to evaluate the specific relationship between each LIWC variable and our sample data compared to population norms. In these models, we included both fixed and random intercepts corresponding to the comedian to account for the nested nature of our data and control for variance arising from random effects such as individual comedian differences. While we considered dimension reduction techniques, these did not yield factors that aligned with the LIWC categories central to our study. We chose to fit separate models for each variable to align with our theoretical framework and to serve as a pre-filtering process, focusing on variables that showed significant differences from the normative data for further analysis.

For each model, we obtained estimates and standard errors. Residuals were scrutinized for normality and linearity through methods such as q-q plots and formal tests like the Shapiro-Wilk test. In cases where deviations from normality were detected (i.e., *p* < .001), the response variable was transformed using a logarithmic or cubic/quadratic root square distribution as appropriate. To detect significant differences between our sample and the population norm for each category, we calculated the difference for each LIWC variable and standardized it into a z-score. We then conducted probability analysis on these standardized scores to provide p-values for each difference. We applied a Bonferroni correction to account for multiple comparisons across the LIWC variables. This conservative approach was chosen to mitigate the risk of Type I errors, ensuring that only the most statistically significant findings were highlighted.

In the subsequent step, we focused on predicting show ratings using the variables identified as positively significant in the first step. Gender (male/female) and the year of production of the show were additionally considered as potential moderators and incorporated into the model. Comedian-specific variability was accounted for as a random effect, while the scaled number of voters was included as a weight in the model. While these models allow us to predict IMDb ratings based on the language used, ‘prediction’ here refers to statistical estimation, indicating that given specific linguistic elements, certain ratings are likely to occur. However, these models do not establish that the language used directly causes the ratings. Instead, they highlight associations that can inform our understanding of how different linguistic features may correlate with audience reception.

All statistical analyses were performed using R software [29], with LMMs constructed using the *lmertest* package and maximum likelihood estimation [30]. Our findings were presented with estimates, standard errors, t-values, confidence intervals, p-values, and effect sizes. *Cohen’s d* was used to estimate effect sizes, calculated as the difference in means divided by the pooled standard deviation, following the approach outlined by Morris and DeShon [31]. Interpretation of *Cohen’s d* values followed conventional guidelines, with values of 0.2, 0.5, and 0.8 considered small, medium, and large effects, respectively [32].

## Results

We examined linguistic differences between our sample of comedians and norms from a broader population using LIWC categories as outlined in Table 1. These categories were selected based on insights from the literature, emphasizing cognitive, social, and affective processes involved in humor generation. We anticipated that comedians would exhibit significantly higher scores in these categories compared to general population norms. Utilizing LMMs, we analyzed each LIWC category individually, treating the intercept as a fixed effect and the comedian as a random effect. The results uncovered significant differences between our sample of comedians and the general population norms across several LIWC categories, particularly those associated with social and affective processes.

Table 2 provides a summary of the estimates, standard errors, norm values, differences from the norms, standardized differences, and corresponding p-values for each LIWC category. Negative differences indicate LIWC categories where comedians exhibited higher scores compared to the general population, while positive differences indicate lower scores among comedians. The significance of these differences was confirmed through p-values obtained from LMMs, which compared comedian scores to population norms across various LIWC categories. Only categories that reached significance are displayed in the table.

**Table 2:** Differences between comedians and the population norms in selected LIWC categories

LIWC Category	Intercept	Std. Error	Norm	Difference	Std. diff	*p* value
Cognition	12.25	0.19	13.86	1.61	8.55	0.000
All-or-none	1.91	0.04	2.03	0.12	3.50	0.000
Cognitive processes	10.25	0.19	11.72	1.46	7.74	0.000
Tentative	12.25	0.19	2.76	-9.49	-50.42	0.000
Certitude	0.71	0.02	1.05	0.34	19.02	0.000
Differentiation	2.96	0.06	3.61	0.66	10.17	0.000
Positive tone	2.53	0.06	4.01	1.48	23.50	0.000
Negative tone	1.75	0.07	1.07	-0.67	-10.10	0.000
Emotion	1.54	0.04	2.26	0.71	17.46	0.000
Positive emotion	0.80	0.02	1.59	0.79	34.45	0.000
Negative emotion	0.61	0.02	0.49	-0.12	-5.03	0.000
Anxiety	0.34	0.01	0.07	-0.27	-25.06	0.000
Anger	0.40	0.01	0.11	-0.29	-22.52	0.000
Sadness	0.27	0.01	0.06	-0.21	-19.29	0.000
Swear words	1.00	0.05	0.29	-0.71	-14.17	0.000
Social processes	15.25	0.21	11.32	-3.93	-18.87	0.000
Social behavior	3.74	0.09	2.74	-1.00	-11.37	0.000
Prosocial behavior	0.64	0.01	0.33	-0.32	-24.64	0.000
Interpersonal conflict	0.53	0.02	0.09	-0.43	-25.36	0.000
Moralization	0.50	0.01	0.11	-0.39	-26.96	0.000
Communication	1.88	0.07	1.58	-0.30	-4.52	0.000
Social referents	11.54	0.19	8.49	-3.05	-15.71	0.000
Family	0.85	0.03	0.30	-0.55	-17.93	0.000
Friends	0.42	0.02	0.24	-0.17	-11.16	0.000
Female references	1.48	0.08	0.75	-0.73	-9.61	0.000
Male references	2.14	0.07	1.07	-1.07	-14.72	0.000

Note: Intercepts and standard errors result from separate LMMs, with the selected LIWC category as the response and comedians as random effects. Norm values were retrieved from the LIWC manual [28]. Differences were obtained by subtracting intercepts from norms, standardizing them by dividing by the standard error, and calculating p-values. Negative differences indicate LIWC categories where comedians exhibited higher scores compared to the general population

When examining differences among cognitive elements in the text, our sample displayed significantly lower frequencies for several categories related to the overarching domain, with the exception of the category *Tentative* (e.g. “if”, “or”, “any”, “something”). Notably, categories such as *Insight*,* Causation*,* Discrepancy*, and *Memory* did not exhibit any significant differences between our sample and the population.

In terms of words related to Affect, although the overarching category *Affect* did not reach significance compared to the general population, comedians used terms related to negative affect more frequently. These included *Negative tone*,* Negative emotion*,* Anxiety*,* Anger*,* Sadness*, and *Swear words*.

Nearly all categories related to the *Social* domain reached significance when comparing the two groups, except for *Politeness* (e.g. “thank”, “please”, …). In all these categories, comedians displayed significantly higher frequencies, highlighting the prevalence of social elements in humor.

These findings highlight the distinctive linguistic patterns exhibited by comedians during humor production, primarily characterized by social and affective processes. The identified LIWC categories with significant negative differences served as the basis for the subsequent analysis, where they were included as predictors in a model aimed at explaining ratings of the comedy shows.

Table 3 offers a comprehensive overview of model estimates, including standard errors, degrees of freedom, and test statistics (t-values). The significance of predictors is indicated by both p-values and confidence intervals, while the effect size is delineated by *Cohen’s d* statistics. Predictors with positive estimates improve the rating of the show, while those with negative estimates worsen it. All predictors are presented in the table.

**Table 3:** Selected LIWC categories predicting standup comedy ratings

LIWC Category	Estimate	Std. Error	Dfs	t value	CI (low)	CI (high)	Cohen’s d	*p* value
(Intercept)	5.01	0.61	131.91	8.16	3.80	6.21	8.16	0.000
Tentative	-0.09	0.14	131.89	-0.65	-0.36	0.18	-0.65	0.516
Negative tone^*^	0.46	0.21	116.44	2.25	0.06	0.86	2.25	0.026
Negative emotion^**^	1.56	0.51	123.31	3.06	0.56	2.56	3.06	0.003
Anxiety^*^	-1.81	0.74	121.94	-2.43	-3.27	-0.35	-2.43	0.017
Anger^***^	-2.44	0.61	115.48	-4.00	-3.64	-1.24	-4.00	0.000
Sadness^***^	-3.95	0.93	116.20	-4.27	-5.77	-2.14	-4.27	0.000
Swear words^*^	-0.17	0.07	131.42	-2.49	-0.31	-0.04	-2.49	0.014
Social processes^***^	1.50	0.32	118.42	4.65	0.87	2.12	4.65	0.000
Social behavior^***^	-1.22	0.34	124.83	-3.58	-1.90	-0.55	-3.58	0.000
Prosocial behavior	0.12	0.39	119.23	0.30	-0.65	0.88	0.30	0.766
Interpersonal conflict	-0.45	0.37	115.81	-1.23	-1.17	0.27	-1.23	0.222
Moralization^***^	-1.93	0.42	116.05	-4.61	-2.75	-1.11	-4.61	0.000
Communication	-0.12	0.22	120.94	-0.56	-0.56	0.31	-0.56	0.576
Social referents^***^	-1.42	0.32	117.69	-4.41	-2.05	-0.79	-4.41	0.000
Family	-0.04	0.13	131.93	-0.28	-0.30	0.23	-0.28	0.776
Friends^*^	-0.84	0.37	120.50	-2.26	-1.57	-0.11	-2.26	0.026
Female references^**^	0.28	0.09	118.70	3.21	0.11	0.45	3.21	0.002
Male references	-0.01	0.09	122.63	-0.14	-0.19	0.17	-0.14	0.888
Gender (male)^***^	0.85	0.19	55.04	4.39	0.47	1.23	4.39	0.000
Production year^***^	-0.35	0.04	123.20	-8.08	-0.43	-0.26	-8.08	0.000

Note: Estimates, statistics, and effect sizes for significant predictors in ratings in the model (*p value < .05, **p value < .01, ***p value < .001). Beta coefficients are unstandardized

As anticipated, male comedians garnered higher ratings compared to their female counterparts. Additionally, the year of production of the show proved to be relevant, with more recent shows generally receiving lower ratings than earlier ones. Regarding LIWC categories, no predictors of *Cognition* could explain changes in the rating, as *Tentative* was found to be not significant. In the *Affect* domain, while *Negative tone* and *Negative emotion* significantly improved the ratings, the presence of words related to *Anxiety*, *Anger*, *Sadness*, and *Swear words* resulted in lower ratings. Mixed effects were also observed in the *Social* domain. While the overarching category positively impacted the ratings, along with Female references, words falling under the categories of Social behavior, *Moralization*,* Social referents*, and *Friends* significantly worsened the ratings.

## Discussion

The study set out to explore the linguistic differences inherent in both the creation and reception of humor, with the ultimate goal of deciphering the underlying mechanisms of what constitutes humor and what elicits laughter, specifically from a linguistic perspective. To accomplish this objective, we obtained transcripts of stand-up comedy performances and subjected the text to automated analysis using a dictionary-based approach. This methodology allowed us to uncover several pivotal insights that shed light on the humor strategies utilized by comedians.

In our initial analysis, we compared the linguistic structures of stand-up comedy transcripts with those of broader speech transcriptions, focusing on key dimensions: Cognition, Affect, and Social processes. These dimensions were chosen based on their widespread presence in humor literature. Interestingly, while cognitive elements were scarcely present, affective and social markers were significantly more prominent in stand-up comedy transcripts. Particularly noteworthy were the heightened frequencies of negative affect and emotions among comedians, including the use of swear words. This observation aligns with established humor theories, which posit that humor often serves as a means of addressing taboo topics and relieving emotional tensions. Moreover, these elements play a crucial role in capturing and maintaining audience attention, thus justifying their prevalence in comedic discourse.

Delving deeper into the Social domain, our analysis revealed a greater frequency of social markers in comedy transcripts across all subcategories. This observation aligns with the idea that humor often involves social interactions and dynamics, a key aspect of the superiority theory of humor, which posits that people find humor in situations where they feel superior to others. Additionally, the abundance of social references aligns with theories emphasizing the role of referencing others in humor creation [33, 34]. Notably, BVT further elucidates how humor arises when there is a perceived distance between the subject matter and the audience, suggesting that comedic content is often perceived as funny when it does not directly impact the audience. Stand-up comedy shows typically adhere to a familiar narrative format, often structured around a series of anecdotes and experiences shared by the artist. This format underscores the interpersonal nature of comedy performances, where comedians draw on personal experiences and interactions with others to engage and entertain their audience. Although the associations observed between linguistic markers and humor appreciation might suggest intriguing patterns that align with various humor theories, it is crucial to understand that these findings serve as a platform for further research into how specific language use might influence or reflect comedic effectiveness but do not establish direct causal links.

After identifying the prevalent linguistic categories among comedians, we proceeded to investigate how language might influence the appreciation of humor, as indicated by ratings of the shows. Before analyzing the impact of linguistic categories on ratings, we first examined whether gender and production year influenced show ratings. Historically, male comedians have held a more prominent position in the comedy industry compared to their female counterparts [35, 36]. This gender disparity is reflected in our findings, as shows by female comedians consistently received lower ratings, underscoring their persistent challenges in garnering recognition and appreciation for their work [37, 38]. This trend may be influenced by societal biases and stereotypes that affect audience perceptions of humor. For example, audiences may have preconceived notions about what is considered ‘funny,’ often favoring traditional or male-dominated comedic styles. Therefore, topics discussed by female comedians may differ from those typically addressed by male comedians, which might not align with some audience expectations or preferences. Additionally, our analysis revealed that newer shows tended to receive lower ratings compared to earlier ones. This trend could be due to several factors. Firstly, IMDb ratings are dynamic and can change over time as more viewers watch and rate the shows. Older shows have had more time to accumulate ratings, which might lead to a more stable and often higher average rating. Secondly, older shows may have been seen, reviewed, and discussed more extensively, leading to greater exposure and potentially higher ratings. Finally, while it is possible that newer generations of comedians face challenges in surpassing the pioneering works that have inspired them, audience nostalgia for groundbreaking performances of earlier comedians may also play a role [39].

While both negative emotion and negative tone had a positive influence on ratings, indicating that their presence enhanced the overall enjoyment of the shows, negative mood words such as anxiety, sadness, and anger had a detrimental effect on stand-up comedy ratings. This divergence in impact suggests a varied relationship between different manifestations of negative affect and humor appreciation. One possible explanation for this disparity is that the presence of anxiety-related words may hinder humor appreciation by creating persistent tension in the shows, detracting from the audience’s ability to fully engage and enjoy the comedic experience. In contrast, a few negative experiences or expressions may serve to add depth and complexity to the narrative, enhancing the overall comedic effect. However, an overwhelming presence of negative mood may intensify the development of the story in a dramatic manner, ultimately diminishing audience appreciation. From a narrative standpoint, the strategic use of negative emotion and expression may contribute to the comedic plot by providing contrast and highlighting the absurdity of certain situations. However, an excessive focus on negative mood may overshadow the comedic elements, leading to a less enjoyable viewing experience for the audience [40]. This is further validated by the disbalance in the LIWC dictionary entries, where ‘negative emotion’ contains 618 entries compared to the combined 435 of its subcategories, which in turn suggests that the broad category encompasses a more diverse array of expressions, further contributing to these divergent effects.

Our analysis also revealed significant predictive power of categories within the Social domain on show ratings, both positively and negatively impacting audience appreciation. Notably, moralization emerged as a factor with a detrimental effect on ratings, suggesting that politically correct content may fail to resonate with audiences. This finding aligns with the notion that provocative or boundary-pushing behavior can attract attention and enhance the visibility of entertainers, even if it may not always align with prevailing social norms [41, 42]. Conversely, the use of social referents, particularly female references such as “she,” “her,” “girl,” and “woman,” was associated with improved ratings. This may suggest that comedians strategically reference individuals outside their own social group as a means of engaging the audience, which could align with elements of the superiority theory. However, it should not be interpreted as conclusive evidence of superiority theory at play, it rather highlights a potential trend. By leveraging social referents, comedians may effectively establish in-group/out-group dynamics, challenge societal norms, and enhance the overall comedic experience for their audience [43].

## Limitations

Several caveats must be considered when interpreting this study’s findings, primarily related to the software used and the study design. The “words as attention” paradigm, as discussed by Boyd and Schwartz [44], provides a descriptive rather than explanatory approach. This reductionistic model limits a comprehensive understanding of how verbal behavior reflects psychological processes, as it relies solely on single-word counts and their categorization. Additionally, this paradigm does not contextualize word embeddings, potentially leading to data misinterpretation. For instance, Rouhizadeh and colleagues [45] demonstrated that the psychological meaning of pronouns can change depending on the discourse context. To address these limitations, future studies could incorporate topic modeling and contextual word embeddings to gain a more refined understanding of the language-psychological processes relationship.

Another limitation of this study is the use of IMDb ratings as a dependent variable. IMDb ratings are dynamic and can change over time as more viewers rate the shows or as cultural and social contexts shift. Therefore, the ratings used in this study represent a snapshot in time and may not fully capture the long-term reception or cultural impact of the stand-up performances. To account for variations in the number of voters, we standardized the ratings, ensuring that comparisons between shows are consistent. Nonetheless, older shows may have accumulated more ratings over time, leading to greater stability and higher average ratings. Additionally, audience nostalgia for pioneering works could also influence this trend.

Other limitations stem from the study design, such as the sample size. The dataset predominantly sourced from a single platform is not evenly distributed across comedians. Some comedians have only two transcripts, while others have five, which could introduce potential biases. Future studies should address these limitations by expanding the sample size. In addition, the use of LIWC normative data may not provide an ideal comparison for stand-up comedy shows. The language used in comedy performances differs significantly from that in other contexts like conversations, blogs, or social media, which are inherently more interactive or casual. These differences could introduce confounds, as each context has its unique language patterns and purposes. Future studies should consider using more contextually similar data, such as non-comedic public speaking events, to enhance the robustness of linguistic comparisons in humor research. Lastly, while we applied a Bonferroni correction to address the risk of inflated error rates due to multiple comparisons, we acknowledge that this approach is conservative and assumes independence between tests, which may not fully apply given the inherent correlations among LIWC variables. However, this conservatism aligns with our goal of filtering for the most relevant categories. Future studies might explore alternative methods or targeted analyses to further refine these findings.

Despite these limitations, the present study offers valuable insights into stand-up comedians’ linguistic markers and their association with gender and performance appreciation. Building upon these findings, future research should aim to include larger and more diverse samples across different nations to explore potential cultural variations in humor. By addressing these limitations and expanding the scope of investigation, a more comprehensive understanding of the linguistic strategies employed by comedians can be achieved.

## Conclusions

This study examined the linguistic elements in stand-up comedy, uncovering key insights into humor creation and audience reception. Stand-up comedy transcripts displayed heightened frequencies of affective and social markers, underscoring their significance in humor production. Female comedians consistently received lower ratings than males, reflecting historical gender biases. Notably, recent shows received lower ratings than older ones, suggesting the challenge of surpassing pioneering works. Negative emotion positively influenced ratings, while negative mood words had a detrimental effect, indicating the delicate balance in comedic narratives. Social markers significantly predicted ratings, with moralization negatively impacting them, while the use of social referents, particularly female pronouns, improved ratings. These findings offer valuable insights into the linguistic dynamics of stand-up comedy, informing future research and practice in the comedy industry.

## Electronic supplementary material

Below is the link to the electronic supplementary material.

Supplementary Material 1

Supplementary Material 2

## Data Availability

The datasets used and/or analyzed during the current study, including the Standup comedy shows feature (Table S1) and the LIWC output (Table S2), are available in the supplementary material files provided with this article. These datasets contain the necessary information to interpret and replicate the findings reported in the article.

## Abbreviations

BVT: Benign-violation theory
LMM: Linear Mixed Models
LIWC: Linguistic Inquiry and Word Count
